# Effects of Dispersal and Initial Diversity on the Composition and Functional Performance of Bacterial Communities

**DOI:** 10.1371/journal.pone.0155239

**Published:** 2016-05-16

**Authors:** Yinghua Zha, Mercè Berga, Jérôme Comte, Silke Langenheder

**Affiliations:** Department of Ecology and Genetics/Limnology, Uppsala University, Norbyvägen 18D, 75236, Uppsala, Sweden; Sun Yat-Sen University, CHINA

## Abstract

Natural communities are open systems and consequently dispersal can play an important role for the diversity, composition and functioning of communities at the local scale. It is, however, still unclear how effects of dispersal differ depending on the initial diversity of local communities. Here we implemented an experiment where we manipulated the initial diversity of natural freshwater bacterioplankton communities using a dilution-to-extinction approach as well as dispersal from a regional species pool. The aim was further to test whether dispersal effects on bacterial abundance and functional parameters (average community growth rates, respiration rates, substrate utilisation ability) differ in dependence of the initial diversity of the communities. First of all, we found that both initial diversity and dispersal rates had an effect on the recruitment of taxa from a regional source, which was higher in communities with low initial diversity and at higher rates of dispersal. Higher initial diversity and dispersal also promoted higher levels of richness and evenness in local communities and affected, both, separately or interactively, the functional performance of communities. Our study therefore suggests that dispersal can influence the diversity, composition and functioning of bacterial communities and that this effect may be enhanced if the initial diversity of communities is depleted.

## Introduction

Biodiversity and in particular species richness, is often positively related to ecosystem functioning [1, 2]. Generally, positive effects of species richness on ecosystem functioning may be due to (a) complementarity effects where higher richness increases the likelihood that species with complementary traits are present or (b) selection effects because particularly good ‘functional performers’ are more likely to be found in communities with higher numbers of species [3]. Given that bacteria are key players in ecosystems, the processes that regulate the diversity of bacterial communities and how this in turn affects the magnitude and stability of ecosystem processes are of central interest. Yet, the existence of a relationship between bacterial richness and ecosystem functioning remains unclear, since reports of positive and sometimes no relationships between these two components are found [4–7]. For bacterial communities positive effects of biodiversity on ecosystem process may be blurred if functional redundancy with regard to the investigated function is high, which can be observed in several cases [8, 9].

Natural communities are open systems and consequently local community diversity (richness and evenness) as well as composition are influenced by dispersal and can lead to changes in ecosystem functioning [10–12]. Firstly, dispersal can change species richness and evenness at the local scale [13–16]. Increases in local richness due to dispersal can be a consequence of neutral dynamics, i.e. related to random immigration events of ecologically equivalent species from the regional species pool, which are more likely to occur at high dispersal rates [14]. Related to this, dispersal increases the likelihood that immigrating species can occupy vacant niches at the local scale [15, 17]. However, communities can also become saturated and species richness can even decline when dispersal rates are very high, for example due to higher resource competition and/or dominance of the same regionally superior species at the local scale, which will result in hump-shaped diversity-dispersal relationships [12, 15, 18, 19]. Secondly, also the composition of bacterial communities at the local scale can be influenced by dispersal. It has, for example, been shown that taxa presence and abundance is often to a strong degree influenced by the regional species pool [20, 21]. In this respect, taxa that are regionally abundant are, on average, also abundant at the local scale, at least when local environmental conditions do not differ strongly from each other [22, 23]. In addition to these neutral effects, dispersal can also be limiting and thereby restrict the occurrence of taxa in suitable habitats, or cause mass effects when dispersal rates are very high [24, 25]. Studies that have looked at how dispersal at different rates influences bacterial community composition at the local scale yielded widely varying outcomes. Sometimes rates as low as 0.009% are enough to cause changes [26] whereas other studies did not observe clear effects unless dispersal rates exceeded 40% [12]. It is currently not clear how and why such different results can be obtained. One possibility is that resident communities at the local scale influence the colonisation success of species that arrive into local communities from the regional species pool. This might, for example, be due to priority effects where local species can monopolise available resources [27]. Another possibility is that the effect of dispersal on local communities depends on the initial diversity of a local community, in particular species richness [28, 29]. If initial diversity is low, the colonisation success of dispersers is predicted to be higher. This might be the case because lower diversity offers more empty niche space that can be filled by external colonisers and/or leaves more resources available and thereby reduces competition [17, 28, 30]. In both cases, this could result in communities that are easier to invade. Further, it has also been shown that sampling and complementarity effects increase the invasion resistance in communities with a higher diversity [31]. For bacterial communities it has been demonstrated that the ability of a pathogenic *Escherichia coli* strain to invade soil bacterial communities is higher when the diversity of the native community is lower [32] and that the invasibility of *Pseudomonas fluorescens* model communities is influenced by e.g. taxonomic and genotypic diversity [33, 34]. However, it is currently unknown if similar patterns will arise when bacterial communities are exposed to dispersal by a complex dispersal source instead of specific invasive species.

Here we implemented an experiment where we prepared batch cultures, which differed in their initial bacterial diversity (dilution rate) as well as with regard to dispersal rates from a regional source community. When the communities reached stationary growth phase, samples were taken to determine effects of initial diversity and dispersal on the realised diversity (richness and evenness), community composition, colonization success of OTUs (operational taxonomic units) from the dispersal source and the ‘functional performance’ of bacterial communities. We specifically tested (1) whether increasing dispersal rates lead to an increase in diversity and stronger changes in community composition, (2) whether the recruitment of taxa from a regional source is stronger in communities with low initial diversity and at higher rates of dispersal; and (3) whether high initial diversity and the rate of dispersal increase the functional performance of bacterial communities.

## Material and Methods

### Sampling

Surface water from the mesotrophic lake Lötsjön was sampled on December 14, 2010 and used as ‘local lake community’. To prepare the dispersal source we collected water from two additional lakes (Långsjön and Ekoln) as well as snow during December 2010 and kept them in the dark at 15°C to acclimatise them to the temperatures used during the experiment. No specific permissions were required to sample the lakes and the sampling did not involve endangered or protected species. Cultures from Långsjön and Ekoln and their respective snow-mixture were treated as regional sources and are named as A (Långsjön inoculum + Långsjön medium), B (Ekoln inoculum + Ekoln medium), C (snow mix inoculum + Långsjön medium) and D (snow mix inoculum + Ekoln medium). The environmental characteristics of the lakes are shown in S1 Table. The reason for working with Lötsjön as the ‘local lake’ and a mixture of Ekoln and Långsjön as dispersal source was that water from the latter two lakes, when mixed at an equal ratio, resulted in DOC, total phosphorus and pH levels similar to those in Lötsjön (S1 Table). Hence, this allowed us to manipulate dispersal of cells without major confounding effects of changes in nutrient and DOC concentrations, even though we cannot exclude the possibility that the molecular characteristic of DOC and other environmental factors differed.

### Experimental set-up

To prepare the medium for the cultures, water from each lake was successively filtered through GF/F (pore size 0.7 μm, Whatman) and 0.2 μm membrane filters (Supor®-200, Pall, Life Science). After incubation at room temperature over night, a second filtration through 0.2 μm membrane filters was done and the water was then autoclaved at 121°C for 20 minutes. Sterilised water was kept in autoclaved 5L glass bottles at room temperature overnight for pH calibration after the pH was re-set to its original value using 1M HCl. The medium was kept at 15°C in the dark until further use.

Pre-cultures needed for the main experiment were set up first for the local lake at different diversity levels and for the different regional sources (A, B, C and D). To prepare the inocula, water was filtered through 0.7 μm GF/F filters to remove grazers and subsequently 30 ml of the respective inoculum was inoculated into sterilized lake water (10% inoculation v/v). For the local lake (Lötsjön), the dilution-to-extinction method was used to obtain cultures with different levels of bacterial diversities. This method uses dilution of a diverse community to remove the rare species and thereby creates mixtures differing in species richness [35]. Dilutions (1:10) were done by serially inoculating 4 ml inoculum into 36 ml sterilized Lötsjön media, then dilutions with concentrations of 10^6^, 10^4^, 10^2^ cell ml^-1^ were chosen to create a diversity gradient. Volumes of 30 ml of each dilution were then transferred into 270 ml sterilized Lötsjön medium. The pre-cultures were incubated at 15°C in the dark until stationary phase was reached, i.e. when there was no further increase in bacterial abundance (see details under ‘Bacterial abundance analyses‘). To ensure that the regional communities, low (10^2^), medium (10^4^) and high (10^6^) diversity cultures were at the same growth stages when used to set-up the main experiment, they were prepared at different time points to account for different community growth rates between them.

Water from the stationary phase of the pre-cultures was used to set-up the cultures with the same 10% dilution ratio. Nine replicates of local lake cultures were prepared for each diversity treatment including three dispersal treatments with three replicates each. One day after inoculation from the pre-cultures, dispersal was implemented by exchanging bacteria in the local lake cultures with cells mixed from each regional sources A, B, C, D, under 3 dispersal rates: no dispersal, medium dispersal rate (exchange 5% of total cell number with cells from the regional cultures per day) and high dispersal rate (exchange 10% of total cell number with cells from the regional cultures per day). The different regional sources were mixed at an equal ratio at the respective rate. The amount of cells to be added from the regional source was calculated according to the cell concentration in the local lake cultures, which was determined every day prior to the dispersal. Dispersal was done once a day and lasted for 4 days. After four days of dispersal, all the local lake cultures were incubated for two more days and after a total incubation of 7 days, samples were taken.

### Measurements

#### Bacterial abundance analysis

Samples for total bacterial abundances in the experiment were taken daily during the main experiment to monitor community growth, preserved with formaldehyde at a final concentration of 2%, stained with Syto 13 according to [36] and counted using a flow cytometer (CyFlow® space, Partec, Germany). Intrinsic growth rates (μ) were derived from the slope of the linear regression model within the time period of linear increase, using ln-transformed bacterial abundance values as a function of time. Bacterial abundances in the pre-cultures were determined daily by staining cells with Acridine orange [37], which were subsequently counted using an epifluorescense microscope.

#### Substrate utilization analysis

Biolog Ecoplates (Biolog, Inc., Hayward, CA) were used to assess and quantify the degree of carbon source utilization by bacterial communities [38, 39]. Volumes of 125 μl from the cultures were inoculated into the wells of the plates, which contained 31 different carbon sources besides one blank in triplicate. Once inoculated, plates were placed at 20°C in the dark. Substrate uptake was quantified by measuring the absorbance at 590 nm with a Tecan ultra evolution microplate reader (Tecan, Austria GmbH) 2 or 3 times per day over a total period of 14 days. Average water colour development (AWCD) in the whole plate was used to assess the response to carbon sources in the bacterial community [40]. When AWCD reached a reference absorbance of 0.5 ± 0.2 (after blank subtraction), plate measurements were stopped since this value indicates the largest difference in substrate utilisation among different microbial communities [41]. Carbon sources with absorbance values above 0.25 when AWCD reached the values of the reference were considered as positive substrates [39], i.e. substrates that could be used by the bacterial communities in the respective wells.

#### Respiration rates measurements

Respiration rates were measured as oxygen consumption over time. The measurements were done after the sampling and lasted for seven days until oxygen concentration was stable. Autoclaved glass vials were filled with 10 ml subsamples from each culture and closed leaving no air in the vial. An oxygen microsensor (Microx Tx3, PreSens) was used for the measurements. Respiration rates were calculated as slopes from linear regressions of oxygen concentration against time.

#### Community composition

Samples from pre-cultures and from the end of the experiment were analysed by 454 pyrosequencing. Subsamples of 150 ml from the cultures were filtered onto 0.2 μm membrane filters (Supor^®^-200, Pall Life), and stored at– 80°C until extraction. DNA extractions were done using the PowerSoil DNA Isolation Kit (MO BIO Laboratories, Inc.). The regions V3 and V4 of the 16S rRNA bacterial gene was amplified by using the forward primer 341 (5’-CCTACGGGAGGCAFCAG-3’) and reverse primer 805R (5’-GACTACCAGGGTATCTAATCC-3’) containing the 454 FLX adaptors and a sample-specific multiplex identifier. PCR triplicates were carried out for each sample and mixed. Each 20 μl PCR reaction consisted of 0.25 μM of forward and reversed primer, 1×HF buffer, 200 μM of each dNTP, 0.4 μg/μl BSA, and 0.02 U/μl Phusion polymerase and 1–2 ng of DNA. Reactions were started with an initial denaturation at 98°C for 30 seconds, followed by 25 cycles of denaturation at 94°C for 10 seconds, annealing at 50°C for 30 seconds, and extension at 72°C for 30 seconds. Then a final primer extension at 72°C for 7 minutes was conducted. PCR products were purified and concentrated by using Agencourt® AMPure® XP (Beckman Coulter). Purified DNA was subsequently quantified with Quant-iT™ PicoGreen® dsDNAReagent Kit (Invitrogen) according to manual instructions. Equal amounts of PCR products from each sample were mixed and sent to the Norwegian High-Throughput Sequencing Centre (Oslo, Norway) for pyrosequencing with a 454 GS FLX system (454 Life Sciences) using Titanium chemistry.

#### Sequence processing

A total of 273,725 sequences were obtained from the sequencing facility. Noise removal was performed following the steps described by Quince et al [42] and led to a substantial removal of sequences (S2 Table). Firstly, a quality-check and truncation at 400 bases was performed and each sample was processed with AmpliconNoise [42]. Following, chimeras were removed using Perseus. Operational taxonomic units (OTUs) were defined using complete linkage clustering at a level of 97% sequence identity using FCluster [42]. Finally, the representative sequences of each OTU were blasted using the RDP database. OTUs with hits corresponding to chloroplasts, chlorophytes and Archaea were removed after classification and excluded from further statistical analysis. The sequence data is available from the Center for Biotechnology Information (NCBI) Sequence Read Archive (SRA) under accession number SRP052953. For statistical data analyses, sampling efforts (number of sequences obtained per sample) were normalised with a rarefied subsampling process across the 34 samples. Samples were subsampled at 3,853 sequences with 100 iterations to account for random effects of the subsampling in R using a personalized script (S1 Appendix). Realized, i.e. final richness (S. Obs) and the Shannon index (H) were calculated from the average of each of 100 iterations using the Vegan package in R [43]. Finally, realized evenness (Pielou’s Evenness index, J) was calculated as J = H/ ln S.Obs.

### Statistical analysis

Differences in community composition between samples were analyzed by non-metric multidimensional scaling (NMDS) based on Bray Curtis similarities calculated from relative abundances using the subsampled OTU table of the sequencing data. Moreover, 2-way permutational multivariate analysis of variance (PERMANOVA) was used to test effects of diversity and dispersal on community composition. Both NMDS and PERMANOVA analyses were carried out using the PAST software package [44].

To test the potential recruitment of taxa from the regional source in the dispersal treatments, we determined the proportion OTUs originating from the regional source. Specifically, we determined the proportion of the dominant taxa of the bacterial community (> 0.5% of total reads) present in a culture at the end of the experiment, that were found exclusively in the regional source, but not in the initial inoculum. We chose a threshold of 0.5% to focus on OTUs that were likely to be growing in batch cultures and, hence, also important functional contributors. The number of OTUs above this threshold varied between 2 and 27 in the different cultures and they accounted for > 90% of total reads in all cases. We calculated the expected relative abundance of OTUs originating from the regional source in the cultures according to their relative abundance in the dispersal source and the dispersal rate. We only included OTUs for which relative abundances in the cultures at the end of the experiment were higher than the ones theoretically expected, to only include taxa that were likely to grow in the cultures and can therefore be considered as successful colonists. Two-way analysis of variance (ANOVA) was then used to test how the proportion of OTUs originating from the regional source in the cultures was influenced by initial diversity and dispersal.

Additionally, two-way ANOVA was used to test the following:

1. how initial diversity (i.e. dilution rate) and dispersal rate affected realised diversity, i.e. richness and evenness at the end of the experiment.
2. how initial diversity and dispersal rate influenced functional performance (respiration rates, growth rates and the number of substrates utilised by the communities) as well as maximal attained bacterial abundance (called ‘bacterial abundance’ in the manuscript).

ANOVAs and subsequent Tukey HSD tests (when main factors were significant) were performed on normalized data using the statistical software program SPSS statistic (version 17.0). Data for respiration rates was log transformed and data for the number of utilised substrates was transformed into ranks to fulfill ANOVA requirements.

## Results

### Effects of dispersal and initial diversity on community composition

**Fig 1:**
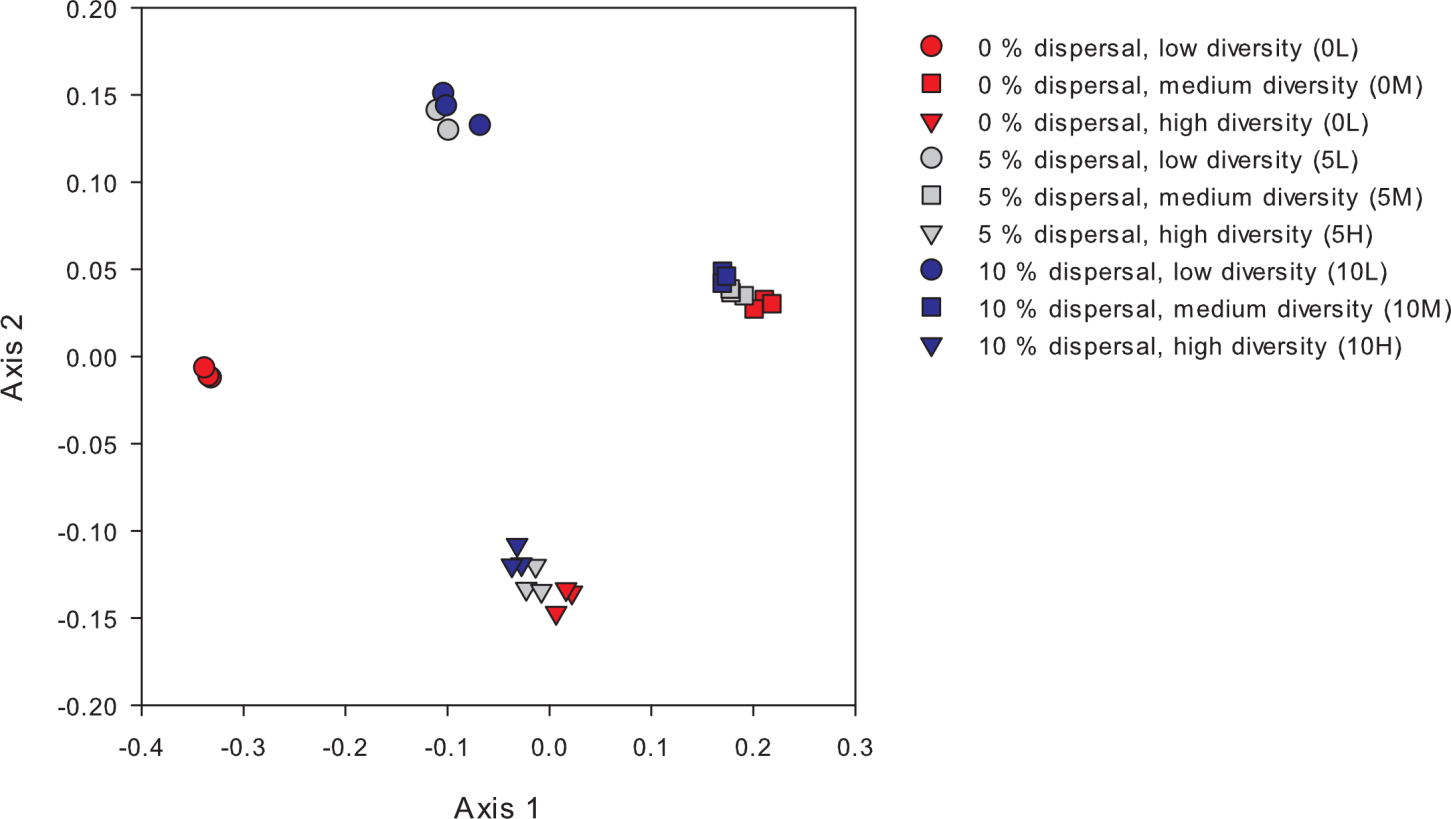
NMDS ordination presenting differences in bacterial community composition between treatments at the end of the experiment. Cultures differed in the level of dispersal: 0% (0), 5% (5) or 10% (10) as well as in their initial diversity: low (L), medium (M) and high (H). K-stress (2D): 0.11.

**Fig 2:**
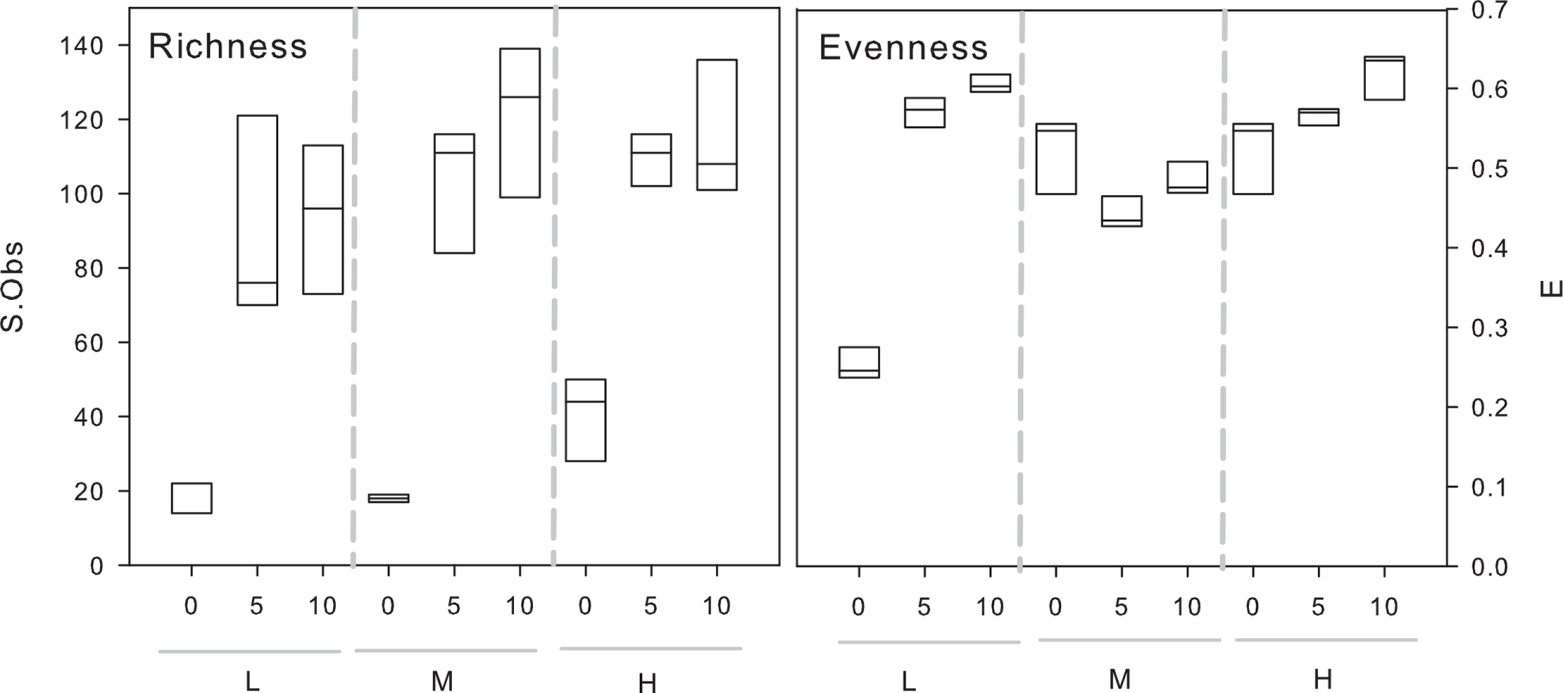
Realised richness (S.Obs) and evenness (E) in cultures. Cultures differed in the level of dispersal: 0%, 5% or 10% as well as in their initial diversity: low (L), medium (M) and high (H).

There were significant effects of initial diversity (dilution rate) as well as dispersal (albeit weaker) on the composition of bacterial communities (2-way PERMANOVA, dispersal: r^2^ = 0.116, p< 0.001, diversity: r^2^ = 0.81, p< 0.001, interaction: r^2^ = 0.073, p< 0.001). NMDS analyses showed that the treatments were separated according to their initial diversity (Fig 1). Moreover, the NMDS showed that communities in treatments with no dispersal and low initial diversity were clearly distinct, whereas the different dispersal treatments of the medium and high initial diversity treatments were much more similar to each other (Fig 1). Dispersal rate and initial diversity had both independent effects on the realised richness (S.Obs) and evenness (E) in the cultures at the end of the experiment, whereas the interaction term was only significant in case of realised evenness (Table 1). Post-hoc tests showed that evenness differed between all levels of initial diversity and dispersal, whereas realised richness was lower in cultures without dispersal and higher in cultures with medium and high initial diversity (Table 1, Fig 2). Realised evenness increased with increasing dispersal rates in the low and high diversity treatment, but not in the medium diversity treatment (Fig 2).

**Table 1:** F- and p-values from two-way ANOVAs testing effects of dispersal and initial diversity (dilution rates) on realised richness (S. Obs), evenness (E), bacterial abundance (Abundance), the number of carbon substrates utilised by the community (# substrates), respiration rates, and growth rates at the end of the experiment. Groupings according to Tukey post-hoc tests for the main effects are shown in brackets, where L, M and H indicate low, medium and high initial diversity and ‘0’, ‘5’ and ‘10’ 0, 5 and 10% dispersal rates, respectively. Significant p-values are shown in bold.

	Dispersal	Diversity	Dispersal × diversity
S.Obs	F = 72.2, p < **0.0001**	F = 4.1, p = **0.034**	F = 32.4, p = 0.60
	(0, 5+10)	(L+M, M+H)	
E	F = 142.6, p < **0.0001**	F = 73.2, p < **0.0001**	F = 31.4, p < **0.0001**
	(0,5,10)	(L, M, H)	
Abundance	F = 1.0, p = 0.384	F = 11.6, p = **0.005**	F = 1.86, p = 0.16
		(L, M+H)	
# substrates	F = 142.7, p < **0.0001**	F = 5.8, p = **0.011**	F = 10.0, p = **0.0002**
	(0, 5+10)	(L+M, H)	
Respiration rates	F = 1.45, p = 0.26	F = 2.19, p = 0.14	F = 4.68, p = **0.007**
Growth rates	F = 4.92, p = **0.02**	F = 8.18, P = **0.003**	F = 2.96, p = **0.048**
	(0+10, 5+10)	(L+M, H)	

### Effects of dispersal and initial diversity on the recruitment of OTUs from the regional source

**Fig 3:**
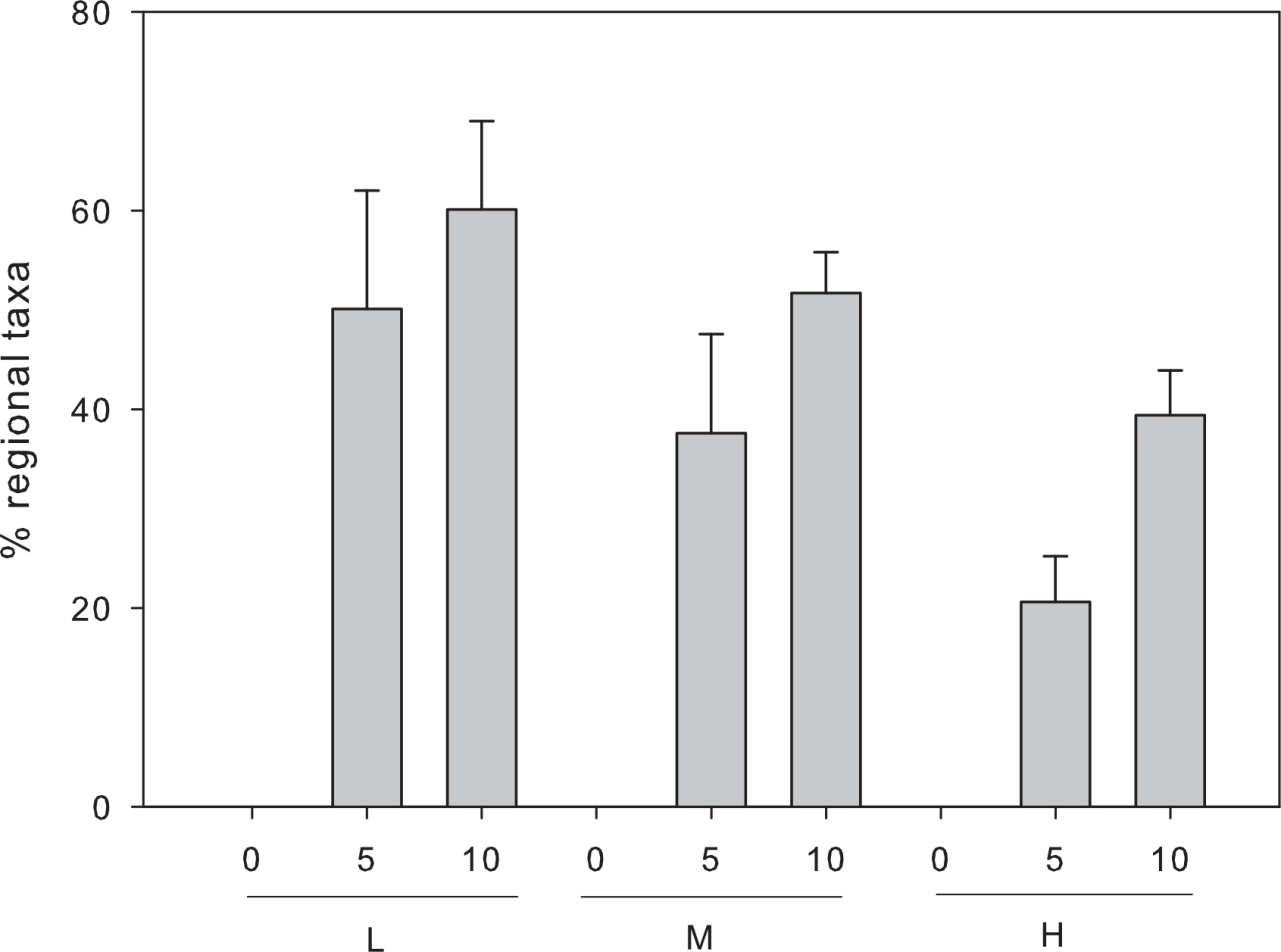
Proportion of dominant taxa (> 0.5% of total reads) in communities at the end of the experiment that could be tracked back to the regional source. Cultures differed in the level of dispersal: 0%, 5% or 10% as well as in their initial diversity: low (L), medium (M) and high (H).

Both, initial diversity (2-way ANOVA, F = 15.1, p = 0.0005) and dispersal rate (F = 14.55, p = 0.002) had significant effects on fraction of species originating from the regional source found among the dominant community members at the end of the experiment, whereas the interaction was not significant (F = 0.45, p = 0.64). The proportion of taxa originating from the regional source community was generally higher in the 10% compared to the 5% dispersal treatment, and was lower in cultures with high initial diversity compared to low diversity, respectively (Fig 3).

### Effects of dispersal and initial diversity on community function and bacterial abundance

**Fig 4:**
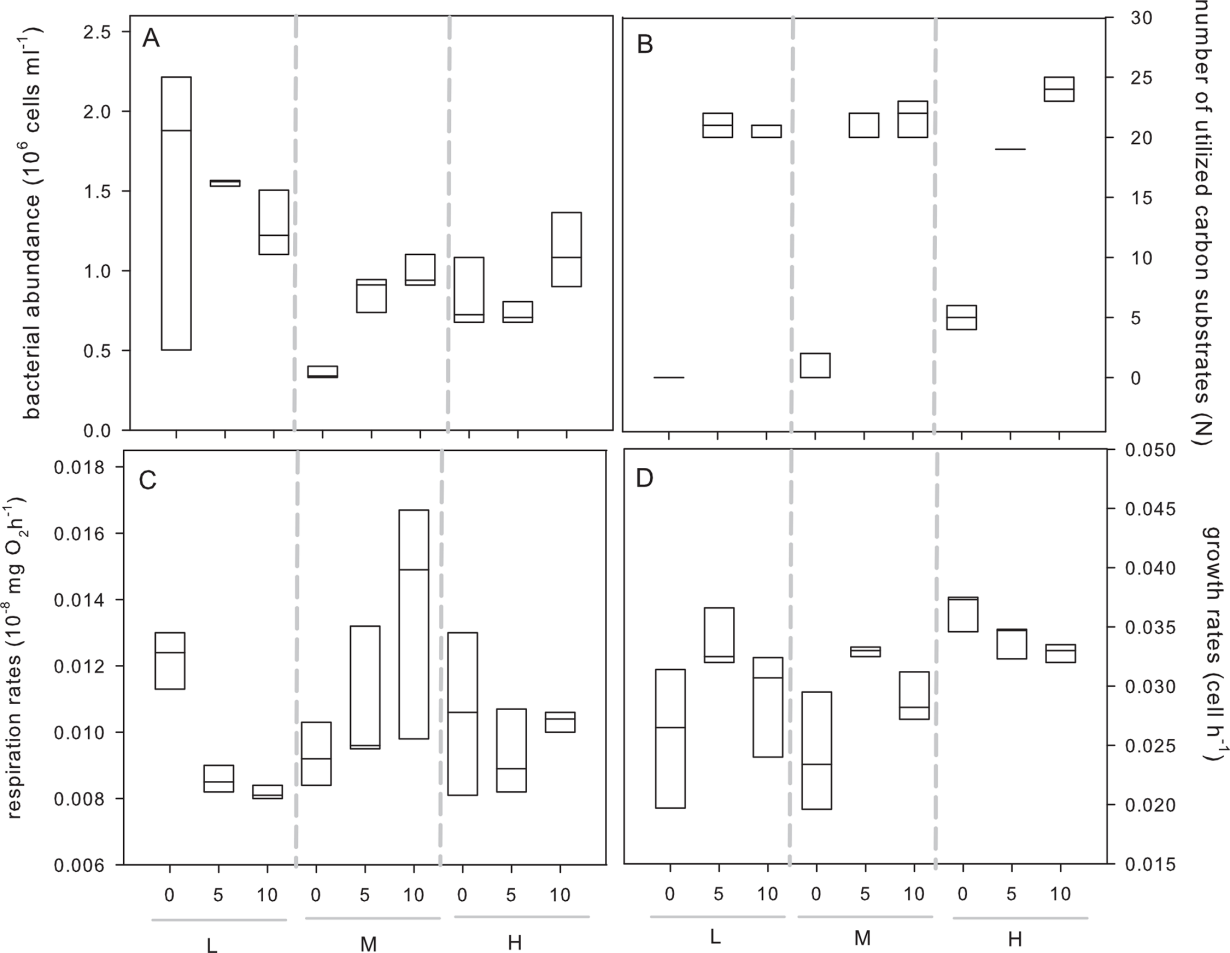
Functional performance of communities at the end of the experiment. Bacterial abundance (A), number of utilized carbon substrates (B), respiration rates (C) and growth rates (D). Cultures differed in the level of dispersal: 0%, 5% or 10% as well as in their initial diversity: low (L), medium (M) and high (H).

Bacterial abundances in the cultures were significantly affected by initial diversity, but not by dispersal rate (Table 1), and were higher in cultures with low compared to medium and high initial diversity (Fig 4A). On the contrary, the number of utilised substrates was significantly affected by both initial diversity and dispersal rate (Table 1) and was, on average, lowest in cultures without dispersal and with low and medium initial diversity (Table 1, Fig 4B). There was also a significant interactive effect of initial diversity and dispersal rate on the number of utilized substrates, which was generally lowest in cultures with low initial diversity and no dispersal (Table 1, Fig 4B). Respiration rates were only significantly affected by the interaction between initial diversity and dispersal rate (Table 1) and decreased with increasing dispersal rates when initial diversity was low, but tended to increase with increasing dispersal rates at medium levels of initial diversity (Fig 4C). Moreover, bacterial growth rates differed significantly from each other depending on initial diversity, dispersal rates and their interaction (Table 1). The significant interaction term showed that growth rates peaked in 5% dispersal treatments compared to no and 10% dispersal treatments in the low and medium initial diversity treatment, but decreased with increasing dispersal rate in the high initial diversity treatment (Table 1, Fig 4D).

## Discussion

The main aim of our study was to test effects of initial diversity and dispersal rate on realised richness and evenness, community composition, colonization success of regional taxa and the ‘functional performance’ of bacterial communities. We could show that dispersal led to an increase in diversity and changes in community composition; that the recruitment of taxa from a regional source was stronger in communities with low initial diversity and at higher rates of dispersal; and that the level of initial diversity and the rate of dispersal also changed the functional performance of bacterial communities. Overall this study suggests that initial diversity affects the sensitivity of a local community to dispersal and that dispersal in general is important because it increases the realised diversity of communities and thereby their functional performance.

Several other studies have investigated how dispersal influences bacterial community composition at the local scale. However, if and at which rates of dispersal effects on the composition of communities at the local scale are seen varies widely between studies. For example, here we saw clear effects at daily dispersal rates of 5 and 10% (with stronger effects at 10%), but sometimes much lower rates are enough to cause changes in community composition [26], whereas other studies did not find effects unless rates were much higher [12]. One possible explanation for these different results could be that—as shown here—effects of dispersal depend on the initial diversity of a community. In support of our hypothesis, we found that the recruitment of taxa from the regional source was higher when initial diversity in communities was low. Our study therefore confirms results of previous theoretical and experimental studies showing that communities with low diversity are more susceptible to invasions [17, 28]. There are also examples that showed that high diversity (both richness and evenness and functional diversity) made bacterial communities less susceptible to invasion by model invaders [32, 33, 45], which was likely to be caused by increased niche space (e.g. more available resources) and reduced competition when invaders were confronted with communities with low diversity. Our results indicate that a similar pattern can also be found if a diverse dispersal source is used because the fraction of taxa from the regional source that could be found among the dominant taxa in the cultures was highest at the lowest initial diversity.

Similarly, realised richness increased when local communities were exposed to dispersal, which is in agreement with theoretical predications [19] as well as findings from previous empirical studies [15, 46–48]. Moreover, there were interactive effects between dispersal rate and initial diversity on realised evenness and community composition. This suggests that interactions between species originating from the local inoculum and colonists from the regional pool had stronger effects on species abundances and dominance than on species richness in the cultures.

Both initial diversity and dispersal affected the functional performance of bacterial communities. Growth rate, respiration rates and carbon utilisation ability generally tended to be slightly higher in cultures with medium and high initial diversity, where also realised richness and evenness were higher compared to those with the lowest initial diversity. This is in congruence with positive diversity–functioning relationships in bacteria [4–7, 49], but also shows that due to high functional redundancy of bacterial communities, effects could not be seen unless the communities were strongly diluted [6, 49, 50]. On the contrary, bacterial abundance was highest in cultures with the lowest initial diversity, which is in contrast to results from dilution-to-extinction studies where cultures with low initial diversity had lower bacterial abundance compared to those with higher diversity [6, 51]. The contrasting pattern we observed in this study may suggest that decreases in species richness and evenness might have reduced negative interactions, such as competition, and instead promoted a more effective resource use, and hence, higher abundance of the most dominant OTUs. Our result that dispersal did not have an effect on bacterial abundance and respiration rates is in congruence with results from Bouvier et al. [51] as well as other studies that show that the carrying capacity of communities in terms of total abundance is often strongly determined by environmental conditions and resource concentration [8, 52]. Dispersal did, however, clearly increase the number of carbon substrates that could be utilised by bacterial communities, potentially because an increased functional diversity and stronger complementarity effects [53, 54] improved their ability to degrade a wider range of carbon substrates. There were, moreover, interactive effects of initial diversity and dispersal on respiration, average community growth rates and, most clearly, the number of carbon substrates utilised by a community. This shows that initial diversity can modify how dispersal changes ecosystem processes, and that the strongest effects can be found if local diversity is low. Hence, effects of dispersal on bacterial functions may not only depend on the environmental context, such as organic matter composition as recently shown [55] but also on differences in initial community diversity.

The diversity levels in our cultures were certainly lower than bacterial diversity in nature, whereas dispersal rates were on the contrary most likely higher, hence it is difficult to extrapolate our results to natural communities and ecosystems. Moreover, it is also likely that the successional state of a community is important and that effects of dispersal are more important at early stages of community development (such as in our batch culture experiment) where communities are generally dominated by fast growing opportunists, which are not characteristic of communities in equilibrium stages [56, 57]. Hence, more studies are now needed to elucidate effects of dispersal at different rates on local bacterial communities and ecosystem processes implemented by bacterial communities under different conditions and over longer periods of time. Nevertheless, our results may still be of general relevance in situations where communities are exposed to strong perturbations (e.g. after droughts and fires) and have to re-assemble from scratch.

In summary, our study suggests that dispersal affects the diversity, composition and functioning of bacterial communities as well as the establishment success of taxa from the regional species pool and that this effect may be influenced by the initial diversity of the communities in some cases. Continued research in this area is now needed to define under which dispersal rates as well as which levels of initial diversity and for which functional parameters these conclusions hold true. This will be important to gain a better understanding about how bacterial diversity in nature is related to rates of ecosystem process at different spatial and temporal scales.

## Supporting Information

S1 Appendix R-script used to subsample the sequences. (R)

S1 Table Environmental characteristics of the three lakes at the time of sampling. (DOCX)

S2 Table Summary statistics of the 16S rRNA 454 sequencing analysis. (DOCX)
