# Supplementary material for: Effects of Dispersal and Initial Diversity on the Composition and Functional Performance of Bacterial Communities
Source: PLoS One. 2016 May 16;11(5):e0155239. doi: 10.1371/journal.pone.0155239 (PMC4868275; doi:10.1371/journal.pone.0155239)
Supplement: S1 Table — (DOCX) [file pone.0155239.s002.docx]

**S1 Table.** Environmental characteristics of the three lakes at the time of sampling (December 14^th^ 2010). Water from Lötsjön was used as the ‘local lakes community’ and the dispersal source was prepared using a 50:50 mix of lake bacterial and snow bacteria grown on medium from lakes Ekoln and Långsjön. Expected values of environmental characteristics in the dispersal sources calculated based on the mixing ratios are included as well.

| **Lake** | **pH** | **DOC (mg/L)** | **Tot-P (μg/L)** |
| --- | --- | --- | --- |
| Lötsjön | 8.06 | 9.7 | 30.8 |
| Ekoln | 7.76 | 13.3 | 43.16 |
| Långsjön | 8.30 | 6.4 | 13.24 |
| Dispersal source | 8.03 | 9.85 | 28.2 |
