# Supplementary material for: Effects of Dispersal and Initial Diversity on the Composition and Functional Performance of Bacterial Communities
Source: PLoS One. 2016 May 16;11(5):e0155239. doi: 10.1371/journal.pone.0155239 (PMC4868275; doi:10.1371/journal.pone.0155239)
Supplement: S2 Table — (DOCX) [file pone.0155239.s003.docx]

**S2 Table.** Summary statistics of the 16S rRNA 454 sequencing analysis.

|  | **Number sequences** | **% lost** | **Range within samples** |
| --- | --- | --- | --- |
| Original* | 275,718 |  | 5,711-10,037 |
| After denoising | 195,549 | 29.1 | 3,939-7,489 |
| After Chimera check (Perseus) | 185,486 | 32.7 | 3,853-7,446 |
|  |  |  |  |
|  | **Number of sequences** | **Number of OTUs** |  |
| Classification | 185,486 | 773 |  |
| After removal of non-bacterial sequences | 185,470 | 766 |  |

*mean sequence length 393.5 bp
